# Multiple Domains in the Kv7.3 C-Terminus Can Regulate Localization to the Axon Initial Segment

**DOI:** 10.3389/fncel.2020.00010

**Published:** 2020-02-04

**Authors:** Louise Leth Hefting, Elisa D’Este, Emil Arvedsen, Tau Benned-Jensen, Hanne Borger Rasmussen

**Affiliations:** ^1^Membrane Trafficking Group, Department of Biomedical Sciences, The Faculty of Health and Medical Sciences, University of Copenhagen, Copenhagen, Denmark; ^2^Optical Microscopy Facility, Max Planck Institute for Medical Research, Heidelberg, Germany

**Keywords:** ankyrin-G, hippocampal neurons, Kv7, KCNQ, FRAP, double-FRAP, STED, nanoscopy

## Abstract

The voltage-gated Kv7.2/Kv7.3 potassium channel is a critical regulator of neuronal excitability. It is strategically positioned at the axon initial segment (AIS) of neurons, where it effectively inhibits repetitive action potential firing. While the selective accumulation of Kv7.2/Kv7.3 channels at the AIS requires binding to the adaptor protein ankyrin G, it is currently unknown if additional molecular mechanisms contribute to the localization and fine-tuning of channel numbers at the AIS. Here, we utilized a chimeric approach to pinpoint regions within the Kv7.3 C-terminal tail with an impact upon AIS localization. This strategy identified two domains with opposing effects upon the AIS localization of Kv7.3 chimeras expressed in cultured hippocampal neurons. While a membrane proximal domain reduced AIS localization of Kv7.3 chimeras, helix D increased and stabilized chimera AIS localization. None of the identified domains were required for AIS localization. However, the domains modulated the relative efficiency of the localization raising the possibility that the two domains contribute to the regulation of Kv7 channel numbers and nanoscale organization at the AIS.

## Introduction

The axon initial segment (AIS) is a unique subdomain in neurons. Located in the beginning of the axon, the AIS is the site where the final summation of input to the nerve cell takes place and where action potentials are initiated. Critical to the regulation of action potential generation, the AIS expresses a very high density of voltage-gated sodium (Nav) channels as well as a repertoire of voltage-gated potassium (Kv) channels (Leterrier, 2016). Intriguingly, the AIS ion channels display unique subcellular localization patterns within the domain. For instance, Nav1.1 displays a proximal AIS localization while Nav1.6, Kv1.1/Kv1.2, and Kv7.2/Kv7.3 channels are predominantly localized to the distal AIS (Van Wart et al., 2007; Battefeld et al., 2014). While some molecular mechanisms underlying the localization of ion channels to the AIS have been unraveled, we currently do not understand how the highly complex organization of ion channels within the AIS is achieved. Further, although of critical importance to regulation of action potential generation, we know very little in regards to the molecular mechanisms controlling individual channel numbers at the AIS.

The Kv7.2/Kv7.3 channel is one of the AIS localized ion channels. It is an epilepsy-linked voltage-gated potassium channel that exerts a strong dampening effect upon repetitive action potential firing (Rasmussen and Trimmer, 2019). Kv7.2/Kv7.3 localization to the AIS requires binding to the scaffolding protein ankyrin G (ankG) that anchors the channels to the betaIV-spectrin/actin cytoskeleton, a mechanism they share with Nav channels (Garrido et al., 2003; Lemaillet et al., 2003; Chung et al., 2006; Pan et al., 2006; Rasmussen et al., 2007). AnkG binding stabilizes Kv7 channels at the AIS, as has also been reported for Nav channels (Brachet et al., 2010; Akin et al., 2015; Benned-Jensen et al., 2016). Whether additional molecular mechanisms contribute to the localization and extreme stability of Kv7.2/Kv7.3 channels at the AIS is currently unknown. It is, however, noteworthy that nanoscale imaging of endogenous Kv7.2 channels has revealed that they do not co-distribute with ankG in nodes of Ranvier (D’Este et al., 2017). Furthermore, AIS ion channels that localize in ankG deficient domains also appear to be very stable at the AIS (Sarmiere et al., 2008; King et al., 2014; Jensen et al., 2017) and for some channels additional microtubule-related mechanisms contributing to their localization at the AIS are starting to emerge (Vacher et al., 2011; Solé et al., 2019). These observations demonstrate that ankG-binding is not the only factor conferring stability to AIS ion channels.

The Kv7.2/Kv7.3 channel is a hetero-tetramer composed of Kv7.2 and Kv7.3 channel subunits. Each subunit is a 6 transmembrane protein with intracellularly localized N- and C-terminal domains. The C-terminal tail is particularly long and contains the ankG binding motif in the distal end (Rasmussen and Trimmer, 2019). In addition, it contains 4 helical domains, helices A-D, that mediate binding to the calcium sensor calmodulin and regulate subunit assembly. Apo-CaM binds to helices A and B and clamps the proximal C-terminus, which facilitates Kv7.2/Kv7.3 channel activation and surface expression (Etxeberria et al., 2008; Liu and Devaux, 2014; Chang et al., 2018). Helices C and D mediate subunit assembly with helix C being required for the tetramerization and helix D regulating the heteromerization between different Kv7 channel family members (Schwake et al., 2006; Howard et al., 2007; Nakajo and Kubo, 2008). As CaM binding facilitates surface expression and, in the case of Kv7.3, promotes heteromeric assembly with Kv7.2, the CaM binding helices can indirectly promote AIS localization of Kv7.2/Kv7.3 channels (Rasmussen et al., 2007; Etxeberria et al., 2008; Cavaretta et al., 2014). It is, however, currently unknown if the long Kv7 C-terminus more directly regulates AIS localization.

In this study, we searched for additional C-terminal domains that regulate Kv7.2/Kv7.3 localization to the AIS. To this end, we applied a chimeric approach to pinpoint regions within the Kv7.3 C-terminus with an impact upon AIS localization and stability. This strategy identified two domains with opposing impact upon AIS localization, a membrane proximal (MP) domain and helix D. While the MP domain reduced AIS localization of Kv7.3 chimeras, helix D increased and stabilized it. The identified domains were not required for AIS localization, but instead regulated the AIS localization efficiency thereby raising the possibility that the domains contribute to the regulation of Kv7 channel numbers at the AIS.

## Materials and Methods

### cDNA Constructs

The hCD4-hKv7.3CT chimera is a fusion between C-terminally truncated human CD4 and amino acids 358–872 of human Kv7.3 corresponding to the entire Kv7.3 C-terminal sequence (CD4: NM_004519; Kv7.3: NP_004510). The cloning of hCD4-hKv7.3CT has been described previously (Gilling et al., 2013). The super-ecliptic pHluorin (SEP)-TAC pcDNA3.1 + vector contains a SEP tag fused to a truncated version of the human interleukin 2 (IL-2) receptor (the TAC antigen) and has been described previously (Benned-Jensen et al., 2016). The same backbone was used when constructing pH-Tomato (pHT)-TAC in pcDNA3.1 + replacing SEP with pHT using *Hin*dIII and *Kpn*I restriction sites. The pHT-tag was kindly provided by the Lab of Richard W. Tsien (Li and Tsien, 2012). The cloning of SEP-TAC-hKv7.3 has been described (Benned-Jensen et al., 2016). The C-terminal sequence of human Kv7.3 was amplified using standard PCR and inserted into *Bam*HI and *Xho*I restriction sites distal to TAC in both the SEP-TAC- and the pHT-TAC vector, thereby generating SEP-TAC-hKv7.3CT and pHT-TAC-hKv7.3CT, respectively. Deletions and mutations in hCD4-hKv7.3CT, SEP-TAC-hKv7.3, SEP-TAC-hKv7.3CT, and pHT-hKv7.3CT chimeras were introduced using site-directed deletion or mutation as described (Liu and Naismith, 2008). The entire coding sequence of all plasmids was verified by sequencing (Macrogen Europe, The Netherlands).

### Neuronal Cultures and Transfections

Dissociated neuronal cultures were prepared from hippocampi dissected out of E18 rat embryos of either sex from timed pregnant Wistar rats (Charles River) as described elsewhere (Kaech and Banker, 2006). All animal use was carried out in accordance with the guidelines of the Danish Veterinary and Food Administration, Ministry of Environment and Food and the protocol approved by the Department of Experimental Medicine at University of Copenhagen. The isolated neurons were grown on an astroglial feeder layer in serum-free Neurobasal medium supplemented with B27, 0.5 mM Glutamax and 10 IU/ml penicillin-streptomycin (Thermo Fischer Scientific). Cells were transfected at 7–9 days *in vitro* (DIV) with a total of 0.8–1.6 μg DNA pr. coverslip (25 mm in diameter) using Lipofectamine2000 (Thermo Fischer Scientific) according to the manufacturers protocol. Neurons were left for expression for 48 h before either fixation or FRAP experiments were performed.

### Immunocytochemistry

Neurons (10 DIV) were fixed in 4% paraformaldehyde in PBS for 20 min or, in the case of pan-Nav1 stainings, for 2 min in 2% paraformaldehyde in PBS followed by 10 min in 100% methanol at −20°C. Blocking of unspecific binding was performed for 30 min with 0.2% fish skin gelatin (Sigma-Aldrich) in PBS for surface-staining or in 0.1% Triton X-100 (Sigma) in PBS (PBST) for total-staining for 30 min at room temperature (RT). Next, neurons were incubated with primary antibodies diluted in 0.2% fish skin gelatin in PBS or PBST for 1 h at RT. Lastly the neurons were incubated with secondary antibodies diluted in 0.2% fish skin gelatin in PBS or PBST for 45 min at RT. Primary antibodies used: mouse anti-CD4 (1:25–1:50 dilution; clone 18–46; Santa Cruz Biotechnology), mouse anti-CD4 (1:50 dilution, MT310, Santa Cruz Biotechnology), rabbit anti-MAP2 (1:100 dilution, H-300, Santa Cruz Biotechnology), mouse anti-pan-Nav1 (1:100 dilution, clone N419/40, RRID:AB_2491098, Neuromab), mouse anti-ankG (1:5, clone N106/65, RRID: AB_10673449, Neuromab), mouse anti-GFP (1:5, clone 4C9, Developmental studies Hybridoma Bank). Primary antibodies were detected using AlexaFluor^®^-conjugated secondary antibodies (Thermo Fischer Scientific). Coverslips were mounted on microscope slides using ProLong Gold or Diamond Antifade Reagent (Thermo Fischer Scientific).

### Confocal Microscopy

Laser-scanning confocal microscopy was performed on upright LSM710 or LSM780 microscopes from Zeiss equipped with argon and helium-neon lasers and a 63x, 1.4 numerical aperture, oil-immersion objective. Imaging settings included a pinhole of 1 airy unit and a pixel format of 1024 × 1024. Line averaging was used to reduce noise. Images in.lsm file format were processed using Zeiss ZEN Black and Blue 2011 software and exported in.tiff format for figures.

### Image Analysis

Images in.lsm file format were analyzed using Fiji (Fiji_Is_Just_ImageJ). The AIS was identified using either pan-Nav or AnkG as marker. Three segmented lines were manually placed in each image; a 90 μm segmented line starting from the soma was drawn along the axon, a 20 μm segmented line was drawn in a dendrite projecting from the soma and a 10 μm segmented line in a region with no cells was included for background subtraction. Fluorescence intensity profiles were extracted from each line and the mean background value subtracted. Mean AIS intensity (the proximal 0–30 μm of the axonal line), mean distal axon intensity (60–90 μm of axonal line) and mean dendrite intensity (0–20 μm of dendrite line) were calculated followed by calculation of AIS/Dendrite and AIS/Distal axon ratios as previously described (Rasmussen et al., 2007).

For quantifications of the AIS start and length of ankG immunolabeling, axonal profiles were smoothed using a 1.45 μm sliding mean and the mean fluorescence intensity in the axonal region distal to the AIS (50–60 μm of axonal line) was subtracted from each value along the axonal profile. AIS start and end positions were identified as the points where fluorescence intensities increased above and dropped below 33% of the max axonal fluorescence intensity, respectively, in line with previous reports (Grubb and Burrone, 2010). AIS length was calculated as the difference between AIS start and AIS end. AIS density of ankG and pan-Nav1 immunolabeling was calculated as the summed fluorescence intensity between AIS start and AIS end, divided by AIS length.

For quantifications of SEP-TAC chimera densities at the AIS, ankG co-immunolabeling was employed to define the AIS start and end positions. As different SEP-TAC chimeras displayed varying levels of both distal axon and somatodendritic labeling, the SEP AIS density was determined as the summed background subtracted surface anti-GFP immunofluorescence signal between the ankG defined AIS start and AIS end positions, divided by AIS length.

### Fluorescence Recovery After Photobleaching (FRAP)

A LSM780 AxioObserver inverted laser-scanning confocal microscope from Zeiss equipped with an incubator and a 63x, 1.4 numerical aperture, oil-immersion objective was used to capture fluorescence from living neurons using a pinhole size of 2.0 airy units. Coverslips with SEP-TAC-Kv7.3 chimeras (single-FRAP) or a combination of SEP-TAC-Kv7.3 and pHT-TAC-Kv7.3 chimeras (double-FRAP) expressing neurons were mounted in an imaging chamber (Warner Instruments) and imaged at 37°C in artificial cerebral spinal fluid (aCSF; in mM: 125 NaCl, 5 KCl, 1 MgCl_2_, 2 CaCl_2_, 10 D-glucose, and 10 HEPES, adjusted to pH 7.4). Single-FRAP experiments were performed at 0.5 Hz for 2 min and double-FRAP experiments were performed at 0.15 Hz for 2 min. The decrease in time-resolution during double-FRAP was necessary to capture two channels at the same time. A circular (ROI) of 5 μm in diameter was placed on the proximal axon and photobleached after 5 frames with high laser power.

### FRAP Analysis

Using Fiji, the mean fluorescence in each frame of the bleached ROI in the AIS was background corrected by subtraction of a ROI placed in a non-cellular region. Furthermore, correction for bleaching was performed by placing a ROI in the distal part of the axon. F(t) corresponds to the fluorescence intensity in the AIS ROI at any given time point corrected for background and bleaching. The normalized recovery (F/F_0_) was calculated using the background and bleach corrected fluorescence intensity F(t) in the first frame (F_B,_ before bleach) and the sixth frame (F_A,_ just after bleach):

$$\frac{F}{F_{0}}=\frac{F\,\left(t\right)-F_{A}}{F_{B}-F_{A}}$$

The relative recovery after 2 min (F_120_/F_0_) was calculated as the mean F/F_0_ value of the last 5 frames.

### STED Microscopy and Data Analysis

Primary hippocampal neurons were prepared from postnatal P0-P2 Wistar rats of either sex (Charles River or Janvier Labs) as previously described (D’Este et al., 2015) and cultured on glass coverslips coated with poly-ornithine (Merck KGaA) and laminin (BD Biosciences). The procedure was conducted in accordance with Animal Welfare Law of the Federal Republic of Germany (Tierschutzgesetz der Bundesrepublik Deutschland, TierSchG) and the regulation about animals used in experiments (Tierschutzversuchsverordnung). For the procedure of euthanizing rodents for subsequent preparation of any tissue, all regulations given in §4 TierSchG are followed. Because euthanization of animals is not an experiment on animals according to §7 Abs. 2 Satz 3 TierSchG, no specific authorization is required. Cells were transfected at 7–8 days *in vitro* (DIV) with 2 μg DNA per coverslip using Lipofectamine2000 (Thermo Fischer Scientific) according to the manufacturers protocol. Neurons were fixed 48 h after transfection with 4% paraformaldehyde in PBS for 20 min. Cells were then quenched with ammonium chloride and glycine (100 mM each) for 5 min, permeabilized with 0.1% Triton X-100 for another 5 min and blocked in PBS supplemented with 1% BSA for 30 min. Both primary and secondary antibody, and nanobody incubations were performed in PBS for 1 h at room temperature or overnight at 4°C. Samples were mounted in Mowiol supplemented with DABCO. Antibodies and nanobodies used: mouse anti-ankG (1:50 dilution, sc-12719, Santa Cruz Biotechnology), rabbit anti-betaIV spectrin (1:250 dilution, a kind gift of Maren Engelhardt, Medical Faculty Mannheim, Heidelberg University), FluoTag^®^-X4-anti-GFP-STAR RED (1:250, N0304-AbRED, Nanotag Biotechnologies). Primary antibodies were detected using AlexaFluor^®^-594 (Thermo Fischer Scientific) or STAR 580 (Abberior) conjugated secondary antibodies.

Samples were imaged on a Abberior STED 775/595/RESOLFT QUAD scanning microscope (Abberior Instruments GmbH) equipped with STED lines at 595 and 775 nm, excitation lines at 355, 405, 485, 561, and 640 nm, spectral detection, and a UPlanSApo 100x/1.4 oil immersion objective lens. Detection windows were set to 580/600–630 nm and 650–725 nm.

Autocorrelation analysis was performed as previously described (D’Este et al., 2017) along 1.5–2 μm long regions selected by the presence of ankG or betaIV spectrin. Briefly, regions of interest were cropped and rotated, to align the vertical axis of the correlation with the axon orientation, maintaining the pixel size. The autocorrelation was calculated with the Matlab R2018a function “xcorr2,” subtracting beforehand the average of the image, to avoid an (uninformative) additive triangular signal in the correlation. The profile along the vertical axis of the correlation image was examined for periodicities. Autocorrelation analyses from different axons were averaged to obtain the mean autocorrelation and standard deviation. Autocorrelation amplitude was defined as the difference between the values at 190 nm (peak) and the average of the values at 95 and 285 nm (valleys) of the 2D autocorrelation functions.

### Statistical Analysis

Data represented originate from neurons collected from at least 3 independent hippocampal neuronal cultures. All data sets were cleaned for outliers in PRISM 8 using ROUT test (*Q* = 0.1%). The cleaned data sets were tested for normal distribution by manual inspection of QQ plots and all were considered normally distributed. As we did not assume standard deviations (SD) to be equal, non-ordinary unpaired *t*-test with Welch’s correction was used when comparing two groups except from double-FRAP analysis in which we employed a paired *t*-test. Non-ordinary Brown-Forsythe ANOVA with Dunnett’s T3 multiple comparisons test was used to compare three or more groups. All box plots display mean (SD) and FRAP curves display standard error of the mean (SEM). Significance is indicated as follows: ^∗^*P* < 0.05, ^∗∗^*P* < 0.01, ^∗∗∗^*P* < 0.001, ^∗∗∗∗^*P* < 0.0001.

## Results

### The Membrane Proximal Domain and Helix D Regulate AIS Localization of CD4-Kv7.3 Chimeras in Hippocampal Neurons

**FIGURE 1:**
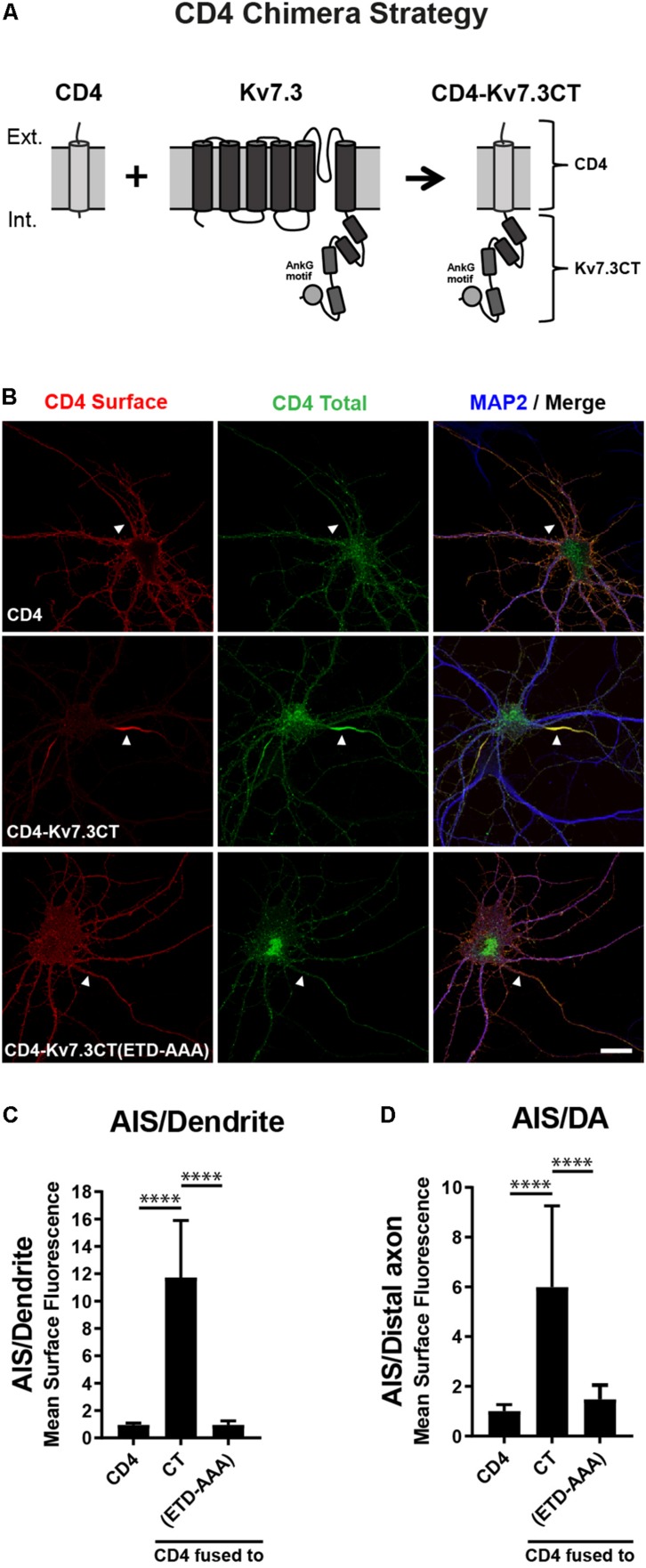
The Kv7.3 C-terminal tail mediates efficient targeting to the AIS. **(A)** Illustration of the CD4 chimera strategy in which a truncated form of the single-pass transmembrane protein CD4 is fused to the C-terminal tail of human Kv7.3. The C-terminus of Kv7.3 contains four α-helical domains, here depicted as cylinders, as well as an ankyrin G (ankG) binding motif, which is depicted as a circle. **(B)** 10 DIV rat hippocampal neurons transiently expressing CD4, CD4-Kv7.3CT or CD4-Kv7.3CT(ETD-AAA). The CD4-Kv7.3CT(ETD-AAA) construct contains point mutations in three essential amino acids of the ankG binding motif (ETD sequence replaced by alanines). Neurons were labeled with CD4 antibody both before and after permeabilization to distinguish surface (red) and total (green) population of CD4-Kv7.3 chimeras. MAP2 was included as a marker of soma and dendrites. White arrowheads point to the AIS positioned in the proximal part of a MAP2 negative neurite. **(C)** Mean CD4 surface fluorescence in the AIS (0–30 μm) versus a dendrite (20 μm), “AIS/Dendrite” ratio for the indicated constructs. Number of neurons analyzed: CD4 (*n* = 14), Kv7.3CT (*n* = 20), and Kv7.3CT(ETD-AAA) (*n* = 16). All data represent the mean (SD). Significances indicated as compared to CT. **(D)** Mean CD4 surface fluorescence in the AIS (0–30 μm) versus the distal axon (60–90 μm), “AIS/Distal axon” ratio for the indicated constructs. Number of neurons analyzed: CD4 (*n* = 15), Kv7.3CT (*n* = 19) and Kv7.3CT(ETD-AAA) (*n* = 19) from 3 to 4 independent experiments. All data represent the mean (SD). Significances indicated as compared to CT. Scale bar, 10 μm.

To search for regions in Kv7.3 with an impact upon AIS localization, we utilized a chimeric approach based on the one-transmembrane reporter protein Cluster of Differentiation 4 (CD4). We replaced the C-terminus of CD4 with C-terminal regions of Kv7.3 (Figure 1A). We first validated the system by expressing CD4 chimeric proteins in cultured hippocampal neurons. Immunocytochemistry demonstrated that the full-length Kv7.3 C-terminus efficiently redirected the surface distribution of the non-polarized CD4 protein to the AIS (Figures 1B–D, CD4-Kv7.3CT). In accordance with published results (Chung et al., 2006), the AIS localization of the CD4-Kv7.3CT chimera was strongly dependent upon an intact ankG binding motif as its mutation resulted in a nearly unpolarized surface distribution of the CD4 chimera [Figures 1B–D, CD4-Kv7.3CT(ETD-AAA)]. The chimeric approach therefore provided an efficient readout in the search for regions important for AIS localization.

**FIGURE 2:**
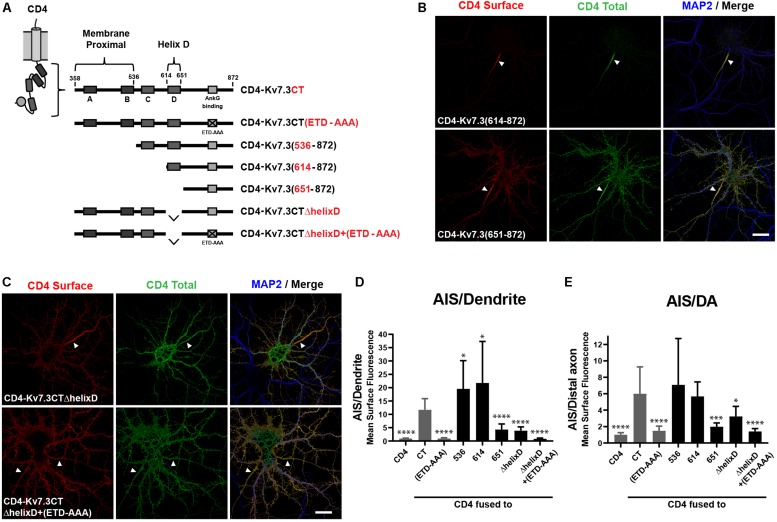
The Kv7.3 membrane proximal domain and helix D regulate localization of CD4 chimeras to the AIS. **(A)** Illustration of CD4-Kv7.3 chimeras investigated in this study. The relative position of the four C-terminal α-helices termed A, B, C, and D as well as the ankG binding motif is indicated. The location of two C-terminal domains, the membrane proximal domain and helix D, is furthermore highlighted. For CD4-Kv7.3(536–872); CD4-Kv7.3(614–872); CD4-Kv7.3(651–872), the numbers refer to the amino acids in Kv7.3 included in the chimeras; CD4-Kv7.3CTΔhelixD contains the C-terminal tail of Kv7.3 with an internal deletion of helix D; CD4-Kv7.3CTΔhelixD + (ETD-AAA) contains an internal deletion of helix D and a mutated ankG-binding motif. In red, the abbreviations used in some figures are indicated. **(B,C)** 10 DIV hippocampal neurons transiently expressing the indicated CD4 chimeras. Neurons were treated as in Figure 1B. **(D)** Mean CD4 surface fluorescence in the AIS (0–30 μm) versus a dendrite (20 μm), “AIS/Dendrite” ratio for the indicated constructs. Number of neurons analyzed: 536 (*n* = 20); 614 (*n* = 26); 651 (*n* = 23); ΔhelixD (*n* = 20); and ΔhelixD + (ETD-AAA) (*n* = 19) from 3 to 4 independent experiments. **(E)** Mean CD4 surface fluorescence in the AIS (0–30 μm) versus the distal axon (60–90 μm) for the indicated constructs, “AIS/Distal axon” ratio. Number of neurons analyzed: 536 (*n* = 22); 614 (*n* = 23); 651 (*n* = 23); ΔhelixD (*n* = 22) and ΔhelixD + (ETD-AAA) (*n* = 19) from 3 to 4 independent experiments. (D,E) CD4, Kv7.3CT, and Kv7.3CT(ETD-AAA) are included as reference values in gray. All data represent the mean (SD). Significances indicated as compared to CT. Scale bars, 10 μm.

Since the ankG binding motif of Kv7.3 is critical for AIS localization, we next constructed chimeras with successive deletions within the C-terminal of Kv7.3, still keeping the ankG binding motif at the distal C-terminus intact (Figure 2A). The resulting chimeras were transfected into cultured hippocampal neurons and their subcellular localization accessed by immunocytochemistry and confocal microscopy. This uncovered the presence of two C-terminal regions with significant impact upon AIS localization of CD4 chimeras. The first domain was revealed by deletion of the proximal 256 amino acids, which resulted in a significant increase in relative AIS localization compared to the CD4-Kv7.3CT chimera [Figures 2B,D,E, CD4-Kv7.3(614–872)]. The increased AIS localization was associated with a strong decrease in the appearance of the chimera outside the AIS. This was most pronounced in the heavily cleared somatodendritic membrane. Additional deletions in the proximal region were attempted to identify the specific proximal domain involved. However, these chimeras did not show significant surface expression when expressed in hippocampal neurons and could not be reliably analyzed for surface distribution. Only when deletions were made distal to helix B, surface expression was recovered and chimera CD4-Kv7.3(536–872) presented the same phenotype as CD4-Kv7.3(614–872) (Figures 2D,E). We were therefore able to narrow down the membrane proximal (MP) domain to the proximal 178 amino acids of the Kv7.3 C-terminus, which includes intact helices A and B known to mediate calmodulin (CaM) interaction (Wen and Levitan, 2002; Yus-Najera et al., 2002).

The second domain with significant impact upon AIS localization was revealed by chimera CD4-Kv7.3(651–872), which contains a deletion of an extra 38 amino acids compared to the strongly AIS expressed CD4-Kv7.3(614–872) chimera. CD4-Kv7.3(651–872) displayed a dramatic and significant decrease in relative AIS enrichment compared to both CD4-Kv7.3CT and CD4-Kv7.3(614–872) [Figures 2B,D,E, CD4-Kv7.3(651–872)]. The relative decrease in AIS enrichment was associated with a stronger appearance of the chimera outside the AIS, primarily in the distal axon, but also in the somatodendritic membrane.

Amino acids 614–651 correspond to helix D, a coiled-coil structure believed to regulate the subtype-specific interaction of different Kv7 channel family members (Schwake et al., 2006). To confirm that deletion of helix D reduces relative AIS localization, we made an internal deletion of helix D in the CD4-Kv7.3CT chimera. Indeed, CD4-Kv7.3CTΔhelixD displayed a surface localization phenotype similar to CD4-Kv7.3(651–872) confirming that deletion of helix D reduces AIS enrichment (Figure 2C, CD4-Kv7.3CTΔhelixD). We finally introduced deletion of helix D in the ankG binding-deficient CD4-Kv7.3CT(ETD-AAA) mutant [Figure 2C, CD4-Kv7.3CTΔhelixD + (ETD-AAA)]. This double-mutant displayed an unpolarized localization at the cell surface similar to CD4 and CD4-Kv7.3CT(ETD-AAA) (Figures 2C–E). All of our microscopy observations were confirmed by quantifications of the AIS/Dendrite and AIS/Distal axon ratios (Figures 2D,E).

### SEP-TAC Chimeras Are Efficient Reporters of Subcellular Localization Signals

**FIGURE 3:**
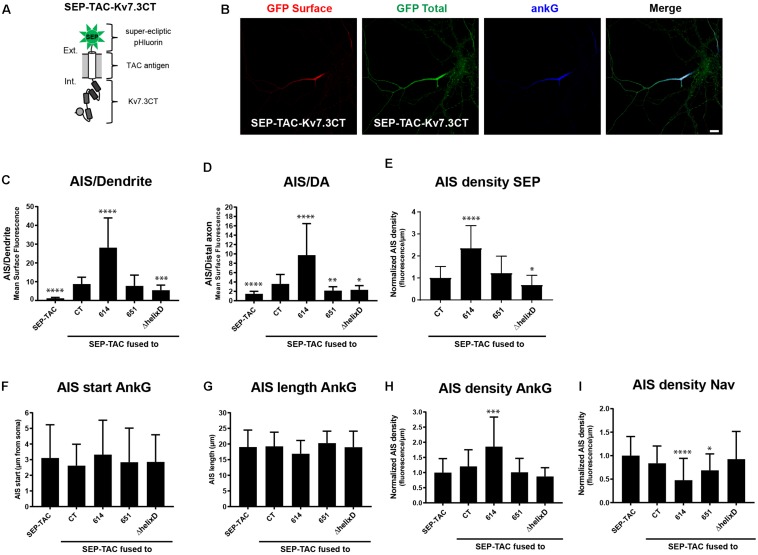
SEP-TAC chimeras are efficient reporters of subcellular localization signals. **(A)** Illustration of the SEP-TAC-Kv7.3CT construct. A truncated single-pass transmembrane protein (IL-2R, known as TAC antigen) fused to the human Kv7.3 C-terminal tail. It is N-terminally tagged with super-ecliptic pHluorin (SEP). **(B)** Confocal image of a 10 DIV hippocampal neuron expressing SEP-TAC-Kv7.3CT. The cell was stained with GFP antibody both before and after permeabilization to distinguish surface (red) and total (green) population of the chimera. Ankyrin-G (ankG) was included as marker of the AIS. Scale bar, 10 μm. **(C)** AIS/Dendrite ratios for the indicated constructs. Number of neurons analyzed: SEP-TAC (*n* = 30), CT (*n* = 30), 614 (*n* = 30), 651 (*n* = 30), ΔhelixD (*n* = 30). Significances indicated as compared to CT. **(D)** AIS/DA ratios for the indicated constructs. Number of neurons analyzed: SEP-TAC (*n* = 30), CT (*n* = 30), 614 (*n* = 29), 651 (*n* = 30), ΔhelixD (*n* = 30). Significances indicated as compared to CT. **(E)** The AIS density of the surface GFP signal in neurons expressing the indicated constructs. All data sets were normalized to the mean of SEP-TAC-Kv7.3CT, which was set to 1. Number of neurons analyzed: CT (*n* = 30), 614 (*n* = 30), 651 (*n* = 30), ΔhelixD (*n* = 30). Significances indicated as compared to CT. **(F)** The AIS start position of ankG immunolabeling in neurons expressing the indicated constructs. Number of neurons analyzed: SEP-TAC (*n* = 30), CT (*n* = 30), 614 (*n* = 30), 651 (*n* = 30), ΔhelixD (*n* = 30). No significant differences in the start position of ankG immunolabeling were observed. **(G)** The AIS length of ankG immunolabeling in neurons expressing the indicated constructs. Number of cells: SEP-TAC (*n* = 30), CT (*n* = 30), 614 (*n* = 30), 651 (*n* = 30), ΔhelixD (*n* = 30). No significant differences in the length of ankG immunolabeling were observed. **(H)** The AIS density of ankG immunolabling in neurons expressing the indicated constructs. All data sets were normalized to the mean of the ankG density in neurons expressing SEP-TAC, which was set to 1. Number of neurons analyzed: SEP-TAC (*n* = 30), CT (*n* = 30), 614 (*n* = 30), 651 (*n* = 30), ΔhelixD (*n* = 30). Significances indicated as compared to SEP-TAC. **(I)** The AIS density of voltage-gated sodium channel (Nav) immunolabeling in neurons expressing the indicated constructs. All data sets were normalized to the mean of the Nav density in neurons expressing SEP-TAC, which was set to 1. Number of neurons analyzed: SEP-TAC (*n* = 29), CT (*n* = 30), 614 (*n* = 29), 651 (*n* = 30), ΔhelixD (*n* = 30). Significances indicated as compared to SEP-TAC. ^∗^*P* < 0.05, ^∗∗^*P* < 0.01, ^∗∗∗^*P* < 0.001, ^∗∗∗∗^*P* < 0.0001.

We next wanted to investigate if the identified regions in the Kv7.3 C-terminal tail influence AIS localization by altering cell surface dynamics. In this context, we recently constructed a fluorescently tagged one transmembrane reporter that can efficiently track chimeras at the cell surface (Humphrey et al., 1993; Benned-Jensen et al., 2016). The chimera is based on a truncated version of interleukin 2 (IL-2), also termed TAC, with super-ecliptic pHluorin (SEP) attached to the N-terminus (Figure 3A). As the fluorescence of this pH-sensitive variant of green fluorescent protein (GFP) is quenched in the more acidic intracellular compartments, it efficiently reports cell surface expression (Miesenböck et al., 1998). We therefore decided to switch to this chimeric reporter and fused Kv7.3 C-terminal regions into the C-terminal domain of the SEP-TAC chimera (Figure 3A).

We first verified that SEP-TAC-Kv7.3 chimeras displayed the same subcellular localization as CD4-Kv7.3 chimeras. Indeed, immunocytochemistry on SEP-TAC-Kv7.3 chimeras expressed in cultured hippocampal neurons demonstrated that they exhibit the same subcellular surface distribution as CD4 chimeras (Figures 3B–D). We also determined the AIS density for the different SEP-TAC-Kv7.3 chimeras. These quantifications demonstrated that deletion of the MP domain increased the AIS density of the chimeras, while deletion of helix D decreased it (Figure 3E).

Overexpression of AIS-targeted proteins has been reported to impact other AIS membrane proteins and the AIS structure itself (Leterrier et al., 2017). We therefore next examined whether similar effects could be observed upon overexpression of SEP-TAC-Kv7.3 chimeras. The position and length of the AIS was unaffected by chimera overexpression as determined by ankG immunolabeling (Figures 3F,G). However, the strongly AIS localized SEP-TAC-Kv7.3(614–872) chimera significantly increased the ankG density and the two chimeras lacking the MP domain, namely SEP-TAC-Kv7.3(614–872) and SEP-TAC-Kv7.3(651–872) significantly decreased pan-Nav1 expression at the AIS supporting the reciprocal interplay between ankG and its membrane partners previously reported (Figures 3H,I; Leterrier et al., 2017). We also wanted to determine whether the chimeras impacted endogenous Kv7 channel density, but found very sparse expression of endogenous Kv7 channels in 10 DIV cultured hippocampal neurons and only in a small subset of cells (data not shown).

### Helix D Regulates AIS Cell Surface Dynamics of Kv7.3 Chimeras

**FIGURE 4:**
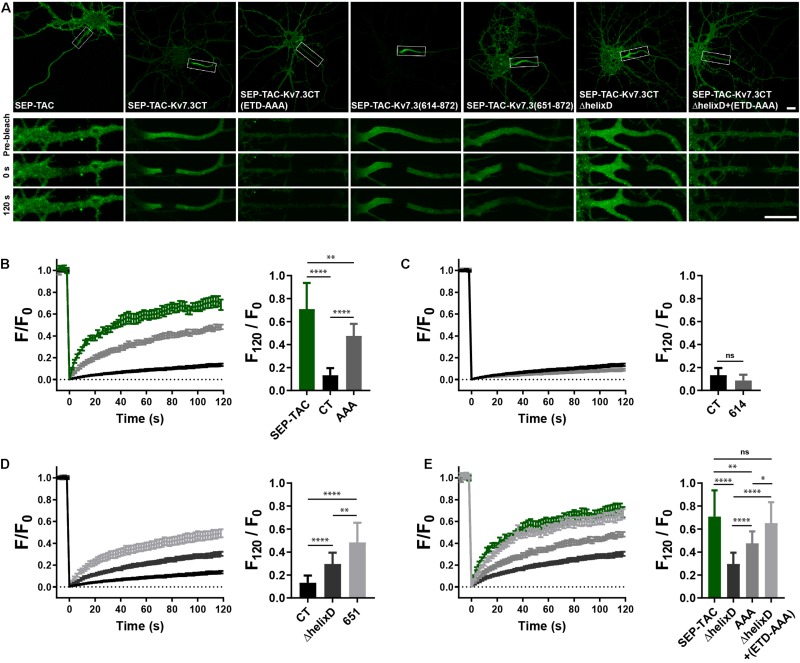
Helix D stabilizes SEP-TAC-Kv7.3 chimeras at the AIS. **(A)** Confocal images of 9–10 DIV live neurons transiently expressing the indicated SEP-TAC chimeras. Fluorescence recovery after photobleaching (FRAP) was performed on the AIS (indicated by the white box in the upper images). Images from the FRAP series (pre-bleach, 0 and 120 s after bleach) is shown below. **(B–E)** FRAP curves showing the quantified fluorescence recovery (F/F0) over the time course of 120 s and the relative recovery of fluorescence at 120 s (F120/F0) calculated as the mean of the last 5 frames from each FRAP series. For reference, SEP-TAC is included again in E, SEP-TAC-Kv7.3CT is included again in (C,D) while the SEP-TAC-Kv7.3CTΔhelixD and Kv7.3CT(ETD-AAA) FRAP curves are included in (E). Number of neurons analyzed: SEP-TAC (*n* = 25), CT (*n* = 30), (ETD-AAA) (*n* = 25), 614 (*n* = 25), 651 (*n* = 21), ΔhelixD (*n* = 26), and ΔhelixD + (ETD-AAA) (*n* = 19) from 3 to 5 independent experiments. FRAP curves show mean ± SEM and column graphs represent mean (SD). Scale bars, 10 μm. ^∗^*P* < 0.05, ^∗∗^*P* < 0.01, ^∗∗∗∗^*P* < 0.0001.

To determine if the MP and helix D domains influence AIS localization by altering cell surface dynamics, we next performed fluorescence recovery after photobleaching (FRAP) on the AIS of neurons transfected with SEP-TAC chimeras. Compared to SEP-TAC, the fluorescence recovery of SEP-TAC-Kv7.3CT was strongly and significantly decreased (Figures 4A,B, SEP-TAC and SEP-TAC-Kv7.3CT). Further, fluorescence recovery was significantly increased upon mutation of the ankG binding site in SEP-TAC-Kv7.3CT demonstrating that the extreme stability is at least partly due to the ankG binding motif [Figures 4A,B, SEP-TAC-Kv7.3CT(ETD-AAA)]. Interestingly, the fluorescence recovery of SEP-TAC-Kv7.3CT(ETD-AAA) did not reach the levels of the SEP-TAC reporter itself suggesting the Kv7.3 C-terminal tail still contained domains stabilizing it at the AIS. We next examined the effect of the MP and helix D domains upon fluorescence recovery. SEP-TAC-Kv7.3(614–872), which lacks the MP domain, displayed a tendency toward reduced fluorescence recovery compared to SEP-TAC-Kv7.3CT though the effect was not significant [Figures 4A,C, SEP-TAC-Kv7.3(614–872)]. In contrast, deletion of helix D significantly and drastically increased fluorescence recovery compared to SEP-TAC-Kv7.3CT. Both SEP-TAC-Kv7.3CTΔhelixD and SEP-TAC-Kv7.3(651–872) displayed significantly increased fluorescence recovery with the largest effect observed in SEP-TAC-Kv7.3(651–872) (Figures 4A,D). In fact, SEP-TAC-Kv7.3(651–872) recovered to the same extent as the ankG-binding deficient SEP-TAC-Kv7.3CT(ETD-AAA) mutant despite the presence of an intact ankG binding motif in the SEP-TAC-Kv7.3(651–872) chimera.

Lastly, we examined the FRAP phenotype of the double mutant with deleted helix D and mutated ankG binding motif. Interestingly, this mutant [SEP-TAC-Kv7.3CTΔhelixD + (ETD-AAA)] displayed an additive FRAP effect when compared to deletion of helix D and mutation of the ankG binding site alone demonstrating that the increased fluorescence recovery in the double-mutant is caused by additive mechanisms (Figures 4A,E). Further, the double mutant reached the same level of fluorescence recovery as the SEP-TAC reporter itself demonstrating that this mutant has lost all its stabilization in the AIS.

### Helix D Regulates the Nanoscale Organization of Kv7.3 Chimeras

**FIGURE 5:**
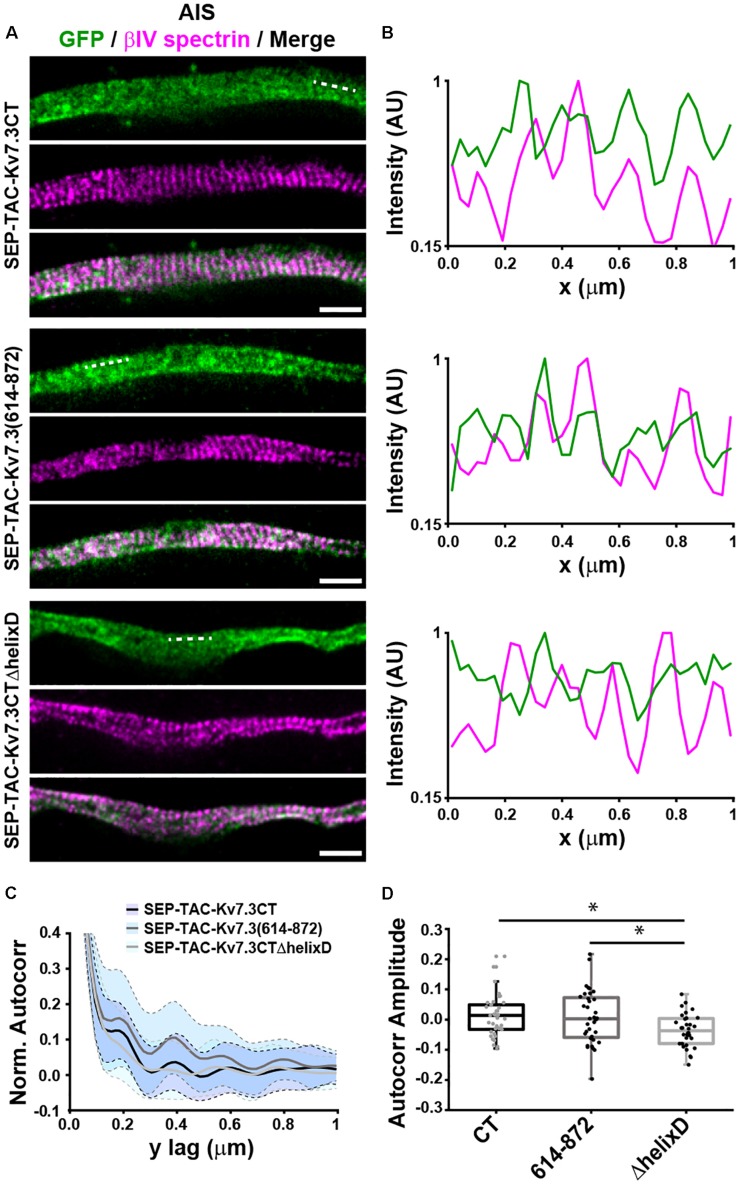
Helix D impacts the nanoscale organization of SEP-TAC-Kv7.3 chimeras at the AIS. **(A)** Superresolution STED images of 9–10 DIV hippocampal neurons expressing SEP-TAC-Kv7.3CT, SEP-TAC-Kv7.3(614–872), or SEP-TAC-Kv7.3CTΔhelixD labeled with a nanobody against GFP and betaIV spectrin to highlight the ∼190 nm periodicity of the subcortical cytoskeleton along the AIS. Single color and merge images are shown. All image data were smoothed with a 1-pixel low pass Gaussian filter. Scale bars, 1 μm. **(B)** Line profile (3 pixels in width) of intensities along the dashed lines indicated on (A) and measured on the raw images. Periodic peaks every ∼190 nm are visible for all the images when analyzing betaIV spectrin. Line profiles of SEP-TAC-Kv7.3CT and SEP-TAC-Kv7.3(614–872) on the same regions show similar peaks, which, however, are not detectable in the case of SEP-TAC-Kv7.3CTΔhelixD. **(C)** 2D average autocorrelation analysis of AIS regions from 9 to 10 DIV hippocampal neurons. The light blue shades indicate the standard deviation of each autocorrelation curve. **(D)** Boxplot of the amplitudes of the 2D autocorrelation functions. The box plot includes the 25th, 50th, and 75th percentile, whereas the whiskers indicate the outliers. Number of axons analyzed in **(C,D)**: SEP-TAC-Kv7.3CT (*n* = 37), SEP-TAC-Kv7.3(614–872) (*n* = 36), or SEP-TAC-Kv7.3CTΔhelixD (*n* = 32) from 3 to 4 independent experiments. ^∗^*P* < 0.05.

The AIS is characterized by the presence of a highly organized cortical scaffold featuring a periodicity of ∼190 nm (Leterrier, 2018). To determine whether the MP and helix D domains impacted the anchoring of SEP-TAC-Kv7.3 chimeras into such scaffold, we performed STimulated Emission Depletion (STED) nanoscopy on neurons transfected with SEP-TAC-Kv7.3CT, SEP-TAC-Kv7.3(614–872), and SEP-TAC-Kv7.3CTΔhelixD (Figure 5). To identify the exact location of the AIS we used betaIV spectrin or ankG staining, which also indicated the regions where the subcortical cytoskeleton is periodic. SEP-TAC-Kv7.3CT and SEP-TAC-Kv7.3(614–872) exhibited a mild periodicity along the AIS (Figures 5A,B). Autocorrelation analysis confirmed the presence of periodic features, showing peaks every 190 nm (Figures 5C,D). On the other hand, SEP-TAC-Kv7.3CTΔhelixD showed no periodic arrangement along the AIS suggesting that deletion of this domain results in a weaker association with the AIS scaffold.

### The FRAP Phenotype of Chimeric ankG-Binding Mutants Can Be Rescued by the Wild-Type Kv7.3 C-Terminal Tail

**FIGURE 6:**
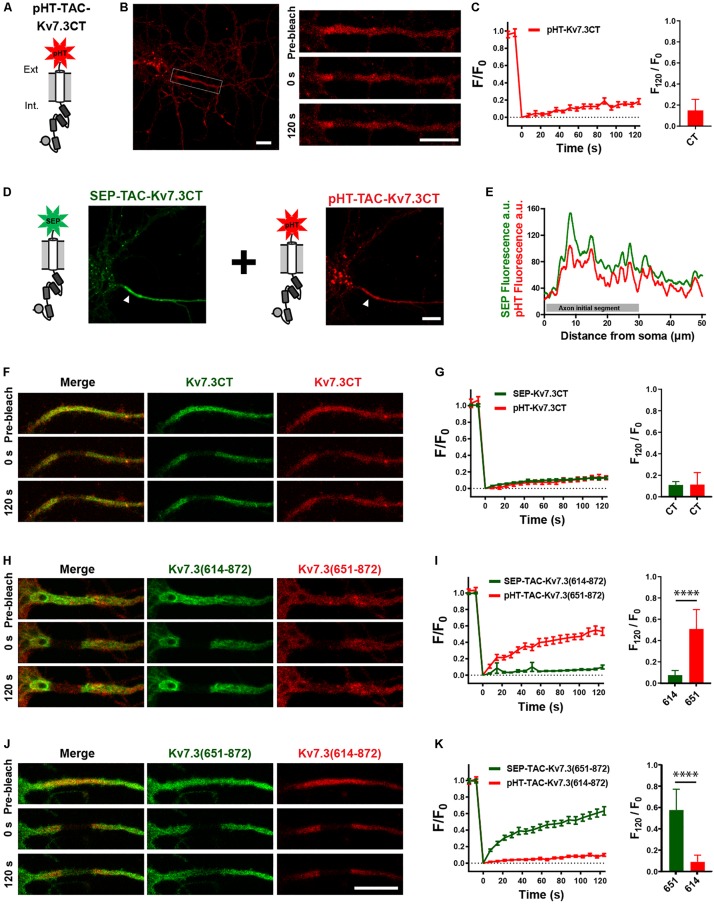
Establishment of a double-FRAP protocol to address TAC chimera assembly. **(A)** Illustration of the pHT-TAC-Kv7.3CT construct in which SEP is replaced by pH-Tomato (pHT). **(B)** Confocal image of 10 DIV live neuron transiently expressing pHT-TAC-Kv7.3CT. The AIS is indicated by a white box. Images from a FRAP series (pre-bleach, 0 and 120 s after bleach) is shown to the right. **(C)** pHT-TAC-Kv7.3CT FRAP curve showing the quantified fluorescence recovery (F/F_0_) over the time course of 120 s and the relative recovery of fluorescence at 120 s (F_120_/F_0_) calculated as the mean of the last 5 frames from each FRAP series. **(D)** Confocal image of a live neuron co-expressing SEP-TAC-Kv7.3CT and pHT-TAC-Kv7.3CT. The AIS is indicated by a white arrowhead. **(E)** Profile from the transfected cell in D of the SEP and pHT fluorescence intensities along a 50 μm axonal line starting from soma. **(F,H,J)** Representative AIS images from double-FRAP experiments (pre-bleach, 0 and 120 s after bleach) in neurons co-expressing the indicated combinations of SEP and pHT chimeras. **(G,I,K)** Double-FRAP curves displaying the fluorescence recovery (F/F_0_) originating from neurons expressing the indicated combinations of SEP and pHT chimeras. Relative fluorescence recovery at 120 s (F_120_/F_0_) is shown to the right. Number of neurons analyzed: pHT-CT alone (*n* = 20); SEP-CT + pHT-CT (*n* = 23), SEP-614 + pHT-651 (*n* = 20), SEP-651 + pHT-614 (*n* = 20), from 3 to 4 independent neuronal cultures. FRAP curves show mean ± SEM and column graphs represent mean (SD). Scale bars, 10 μm.

Helix D has been reported to be a self-assembling peptide that can dictate Kv7 subunit heteromerization (Schwake et al., 2006; Howard et al., 2007; Wehling et al., 2007; Wiener et al., 2008). Further, TAC chimeras containing the Kv7.2 ABCD helices form tetramers in solution, the stability of which depends on helix D (Alberdi et al., 2015). We therefore speculated that the effects of the Kv7.3 helix D could be due to chimera assembly differences. To address this, we established a double-FRAP protocol to examine if TAC-Kv7.3 chimeras co-assemble. We first constructed a pHT-TAC-Kv7.3CT chimera in which SEP was replaced with the pH-sensitive red fluorescent protein pH-Tomato (pHT) (Figure 6A; Li and Tsien, 2012). We next verified that pHT-TAC-Kv7.3CT displayed the same subcellular localization and FRAP phenotype as SEP-TAC-Kv7.3CT. Indeed, pHT-TAC-Kv7.3CT localized efficiently to the AIS (Figure 6B). In addition, bright aggregates were detected, as has previously been reported for fusion proteins containing derivatives of red fluorescent protein (Figure 6B; Landgraf et al., 2012). The aggregates did not, however, hamper the live-cell detection of pHT-TAC-Kv7.3CT at the AIS and subsequent FRAP analysis of pHT-TAC-Kv7.3CT demonstrated that it displayed a FRAP phenotype similar to SEP-TAC-Kv7.3CT (Figures 6B,C). We further validated the system by co-expressing SEP-TAC-Kv7.3CT and pHT-TAC-Kv7.3CT in hippocampal neurons. As expected, pHT-TAC-Kv7.3CT and SEP-TAC-Kv7.3CT displayed similar enrichment at the AIS and similar FRAP phenotypes upon co-expression (Figures 6D–G). Having established that pHT-TAC-Kv7.3CT behaves similar to SEP-TAC-Kv7-3CT at the AIS, we now had a double-FRAP setup that allowed us to surface track two different Kv7.3 chimeras (SEP-TAC- and pHT-TAC-Kv7.3 chimeras) within the same cell.

We first co-expressed SEP-TAC-Kv7.3(614–872) and pHT-TAC-Kv7.3(651–872) as these chimeras display very different FRAP phenotypes. Further, we would not expect these chimeras to co-assemble as pHT-TAC-Kv7.3(651–872) does not contain helices C and D believed to be involved in tetramerization (Schwake et al., 2006). Indeed, simultaneous FRAP analysis of SEP-TAC-Kv7.3(614–872) and pHT-TAC-Kv7.3(651–872) co-expressed in the same neurons demonstrated that the two chimeras retained their respective phenotypes upon co-expression suggesting that they do not co-assemble (Figures 6H,I). A similar result was obtained when inverting the fluorophores verifying that the FRAP phenotype is independent of the fluorophore attached (Figures 6J,K).

**FIGURE 7:**
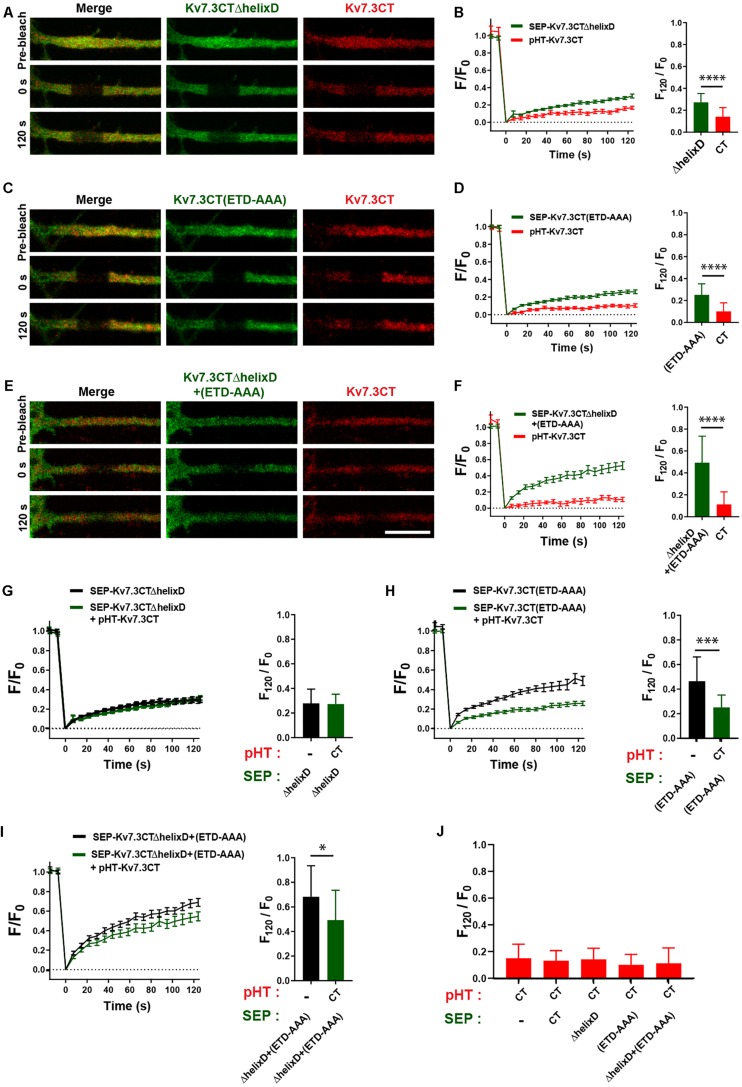
The FRAP phenotype of SEP-TAC-Kv7.3 ankG-binding mutants can be rescued by co-expression of pHT-TAC-Kv7.3CT. **(A,C,E)** Representative AIS images from double-FRAP experiments (pre-bleach, 0 s after bleach and 120 s after bleach) in neurons co-expressing the indicated combinations of SEP and pHT chimeras. **(B,D,F)** Double-FRAP curves displaying the fluorescence recovery (F/F_0_) in neurons transiently co-expressing pHT-TAC-Kv7.3CT and the indicated SEP constructs. To the right the relative fluorescence recovery at 120 s (F_120_/F_0_) for each construct is displayed. Number of neurons analyzed: SEP-ΔhelixD + pHT-CT (*n* = 25); SEP-(ETD-AAA) + pHT-CT (*n* = 25) or SEP-ΔhelixD + (ETD-AAA) + pHT-CT (*n* = 24). **(G–I)** FRAP curves and relative fluorescence recovery at 120 s (F_120_/F_0_) of the indicated SEP constructs expressed alone or co-expressed with pHT-TAC-Kv7.3CT. Number of neurons analyzed: SEP-ΔhelixD alone (*n* = 20); SEP-(ETD-AAA) alone (*n* = 19) or SEP-ΔhelixD + (ETD-AAA) alone (*n* = 22). **(J)** The relative fluorescence recovery at 120 s (F_120_/F_0_) for pHT-TAC-Kv7.3CT expressed alone or in combination with the indicated SEP chimeras. Number of neurons analyzed: pHT-CT alone (*n* = 20); SEP-CT + pHT-CT (*n* = 23), SEP-ΔhelixD + pHT-CT (*n* = 25); SEP-(ETD-AAA) + pHT-CT (*n* = 25), SEP-ΔhelixD + (ETD-AAA) + pHT-CT (*n* = 25). Scale bars, 10 μm.

We next addressed the possible co-assembly of chimeras containing helix C. To this end, we co-expressed pHT-TAC-Kv7.3CT with SEP-TAC-Kv7.3CTΔhelixD, SEP-TAC-Kv7.3CT(ETD-AAA), and SEP-TAC-Kv7.3CTΔhelixD + (ETD-AAA), respectively, and analyzed their FRAP phenotypes with double-FRAP. Upon co-expression, the FRAP phenotype of pHT-TAC-Kv7.3CT differed significantly from the FRAP phenotypes of SEP-TAC-Kv7.3CTΔhelixD, SEP-TAC-Kv7.3CT(ETD-AAA) and SEP-TAC-Kv7.3CTΔhelixD + (ETD-AAA) (Figures 7A–F). However, we noticed that the fluorescence recovery of SEP-TAC-Kv7.3CT(ETD-AAA) and SEP-TAC-Kv7.3CTΔhelixD + (ETD-AAA) appeared to be reduced. We therefore compared the FRAP phenotypes of singly expressed SEP-TAC-Kv7.3CTΔhelixD, SEP-TAC-Kv7.3CT(ETD-AAA) and SEP-TAC-Kv7.3CTΔhelixD + (ETD-AAA) to the FRAP phenotypes observed upon co-expression with pHT-TAC-Kv7.3CT (Figures 7G–I). The presence of pHT-TAC-Kv7.3CT had no significant effect upon the FRAP phenotype of SEP-TAC-Kv7.3CTΔhelixD (Figure 7G). However, pHT-TAC-Kv7.3CT significantly reduced the fluorescence recovery of the two ankG binding mutants, namely SEP-TAC-Kv7.3CT(ETD-AAA) and SEP-TAC-Kv7.3CTΔhelixD + (ETD-AAA), suggesting that the chimeras co-assemble and can do so in the absence of helix D (Figures 7H,I). We finally examined whether the fluorescence recovery profile of pHT-TAC-Kv7.3CT was influenced by the presence of co-expressed SEP-TAC chimeras (Figure 7J). Interestingly, the fluorescence recovery of pHT-TAC-Kv7.3CT was independent of the co-expressed SEP-TAC-Kv7.3 chimeras demonstrating that none of the mutants exert a dominant phenotype over the wildtype C-terminal tail (Figure 7J).

### Helix D Regulates the AIS Density and Cell Surface Dynamics of Kv7.2/Kv7.3 Full-Length Channels by a Mechanism Additive to That of the AnkG Binding Motif

**FIGURE 8:**
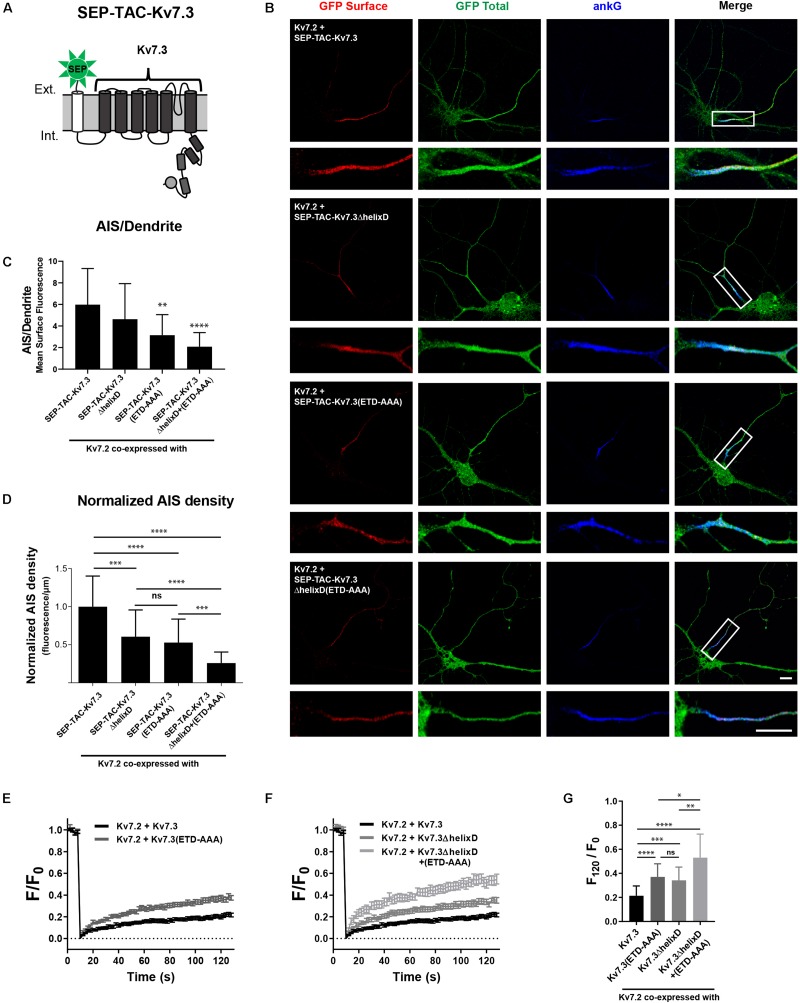
Helix D regulates the AIS density and cell surface dynamics of Kv7.2/Kv7.3 full-length channels by a mechanism additive to that of the ankG binding motif. **(A)** Illustration of the SEP-TAC-Kv7.3 full-length construct. **(B)** Confocal images of 10 DIV hippocampal neurons co-expressing full-length Kv7.2 and the indicated full-length SEP-TAC-Kv7.3 chimeras. Neurons were stained with GFP antibody both before and after permeabilization to distinguish surface (red) and total (green) population of the chimera. AnkG was included as marker of the AIS. The white boxes highlight the AIS, which is magnified below. Scale bars, 10 μm. **(C)** AIS/Dendrite ratios for the indicated constructs. Number of neurons analyzed: Kv7.2 + SEP-TAC-Kv7.3 (*n* = 29), Kv7.2 + SEP-TAC-Kv7.3ΔhelixD (*n* = 30); Kv7.2 + SEP-TAC-Kv7.3(ETD-AAA) (*n* = 29), Kv7.2 + SEP-TAC-Kv7.3ΔhelixD + (ETD-AAA) (*n* = 23). Significances as compared to Kv7.2 + SEP-TAC-Kv7.3. **(D)** The AIS density of the surface GFP signal in neurons expressing the indicated constructs. All data sets were normalized to the mean of Kv7.2 + SEP-TAC-Kv7.3, which was set to 1. Number of neurons analyzed: Kv7.2 + SEP-TAC-Kv7.3 (*n* = 30), Kv7.2 + SEP-TAC-Kv7.3ΔhelixD (*n* = 31); Kv7.2 + SEP-TAC-Kv7.3(ETD-AAA) (*n* = 30), Kv7.2 + SEP-TAC-Kv7.3ΔhelixD + (ETD-AAA) (*n* = 26). **(E)** FRAP curves showing the quantified fluorescence recovery (F/F0) over the time course of 120 s for SEP-TAC-Kv7.3 and the ankG binding mutant SEP-TAC-Kv7.3(ETD-AAA) in neurons co-expressing wild-type Kv7.2. Number of cells analyzed: Kv7.3 (n = 25), Kv7.3(ETD-AAA) (n = 24). **(F)** FRAP curves showing the quantified fluorescence recovery (F/F0) over the time course of 120 s for SEP-TAC-Kv7.3ΔhelixD and the double-mutant Kv7.3ΔhelixD + (ETD-AAA) in neurons co-expressing wild-type Kv7.2. SEP-TAC-Kv7.3 is included again for reference. Number of cells analyzed: Kv7.3ΔhelixD (*n* = 24), and Kv7.3ΔhelixD + (ETD-AAA) (*n* = 23). **(G)** The relative recovery of fluorescence at 120 s (F120/F0) for the indicated constructs, calculated as the mean of the last 5 frames from each FRAP series. Number of cells analyzed: Kv7.3 (*n* = 25), Kv7.3(ETD-AAA) (*n* = 24). Kv7.3ΔhelixD (*n* = 24), and Kv7.3ΔhelixD + (ETD-AAA) (*n* = 23). ^∗^*P* < 0.05, ^∗∗^*P* < 0.01, ^∗∗∗^*P* < 0.001, ^∗∗∗∗^*P* < 0.0001.

SEP-TAC-Kv7.3 C-terminal chimeras represent a simplified system for studying AIS localization of Kv7 channels. We therefore examined whether our observations could be transferred to full-length Kv7 channels. Kv7.2/Kv7.3 channels require co-assembly to localize to the AIS (Chung et al., 2006; Rasmussen et al., 2007). We thus could not make deletions corresponding to the SEP-TAC-Kv7.3(614–872) chimera as it would interfere with channel assembly by removing helix C. However, we deleted helix D, which can regulate heterotetramerization, but is not required for channel assembly (Nakajo and Kubo, 2008). As the backbone, we used SEP-TAC-Kv7.3, a 7 transmembrane chimera that allows surface tracking of full-length Kv7 channels (Figure 8A; Benned-Jensen et al., 2016). We made the following full-length Kv7.3 constructs: SEP-TAC-Kv7.3ΔhelixD, SEP-TAC-Kv7.3(ETD-AAA) and SEP-TAC-Kv7.3ΔhelixD + (ETD-AAA) and co-expressed them with wild-type Kv7.2 in cultured hippocampal neurons. In accordance with previous observations, Kv7.2/SEP-TAC-Kv7.3 channels localized efficiently to the AIS (Figure 8B). Kv7.2/SEP-TAC-Kv7.3ΔhelixD channels also localized at the AIS, demonstrating that the subunits can co-assemble in the absence of helix D. However, they displayed a significantly reduced expression at the AIS of approximately 50% (Figures 8B–D). A similar reduction in AIS localization was observed in channels lacking the Kv7.3 ankG binding motif [Figures 8B–D, Kv7.2 + SEP-TAC-Kv7.3(ETD-AAA)]. Interestingly, the double mutant lacking both helix D and the ankG binding motif displayed an additive effect with a significantly stronger reduction in AIS expression compared to the two single mutants [Figures 8B–D, Kv7.2 + SEP-TAC-Kv7.3ΔhelixD + (ETD-AAA)].

We finally performed FRAP on the AIS of neurons co-expressing full-length Kv7.2/SEP-TAC-Kv7.3 chimeras. In accordance with previous observations, Kv7.2/SEP-TAC-Kv7.3 channels were very stable at the AIS (Figures 8E–G; Benned-Jensen et al., 2016). Further, mutation of the Kv7.3 ankG binding site significantly increased fluorescence recovery as compared to Kv7.2/SEP-TAC-Kv7.3 [Figures 8E,G, Kv7.2 + SEP-TAC-Kv7.3(ETD-AAA)]. Importantly, deletion of helix D also significantly increased fluorescence recovery (Figures 8F,G, Kv7.2 + SEP-TAC-Kv7.3ΔhelixD), and the effect was additive to that of mutating the ankG binding motif as the double mutant displayed a significantly increased fluorescence recovery as compared to the two single mutants [Figures 8E–G, Kv7.2 + SEP-TAC-Kv7.3ΔhelixD + (ETD-AAA)].

## Discussion

Recent research has revealed an unanticipated complex molecular organization of the ion channels at the AIS with individual ion channels displaying unique subcellular localization patterns within the domain (Leterrier, 2016). For several of the ion channels, the major known localization mechanism involves the scaffolding molecule ankG that can impact both ion channel insertion and stability in the AIS (Brachet et al., 2010; Akin et al., 2015; Benned-Jensen et al., 2016). However, ankG is distributed throughout the AIS and ankG binding alone fails to explain the different localizations of AIS localized ion channels. This suggests that other molecular mechanisms operate to modulate ion channel localization at the AIS. In this study, we searched the Kv7.3 C-terminus for regions that regulate AIS localization in addition to the ankG binding motif and identified two C-terminal domains with an impact upon AIS localization of Kv7.3 chimeras. In the process, we developed the SEP-TAC- chimera as an efficient screening tool for peptide sequences modulating localization and surface dynamics of membrane proteins.

The first domain identified encompasses aa 358–535 of the Kv7.3 C-terminus, which contains helices A and B as well as the linker connecting them. Additional deletions from the proximal part of the C-terminal tail proved unsuccessful in further narrowing down the domain as these deletion mutants failed to efficiently reach the cell surface. All of the tested proximal deletions eliminate helix A, which is critical for the binding of apoCaM and our data thus further support that the binding of apoCaM to Kv7 channels is critical for efficient surface expression (Etxeberria et al., 2008; Liu and Devaux, 2014; Chang et al., 2018).

The deletion of the MP domain increased the AIS localization of Kv7.3 chimeras suggesting the domain contains regions that negatively regulate Kv7 localization to the AIS. Interestingly, the deletion had no significant impact on the FRAP phenotype which could indicate that the domain primarily regulates Kv7 trafficking and less so channel anchoring at the AIS. Further, deletion of the domain did not impact the nanoscale organization of the chimeras.

The deleted MP domain mediates interaction with two proteins that could play a role in the effects observed. As mentioned, helices A and B bind CaM, and CaM binding is known to regulate Kv7 surface expression. However, CaM binding promotes Kv7 surface expression rather than inhibiting it (Etxeberria et al., 2008; Liu and Devaux, 2014; Chang et al., 2018). The MP domain binds another interesting candidate, namely the A-kinase anchoring protein (AKAP) AKAP79/150, a scaffold protein for PKA, CaM, PKC and calcineurin (Hoshi et al., 2003; Wong and Scott, 2004; Bal et al., 2010; Zhang and Shapiro, 2016). AKAP79/150 and its anchored pool of PKC negatively regulates Kv7 channels by promoting the muscarinic inhibition of the channels (Hoshi et al., 2003). While AKAP79/150 has only been reported to regulate Kv7 currents and not channel trafficking, AKAP79/150 is known to regulate the endocytic trafficking of other channel targets and a similar effect could be envisioned for Kv7 (Bhattacharyya et al., 2009; Lin et al., 2011; Li et al., 2013). The exact binding site for AKAP79/150 in the proximal tail has not been determined. It would certainly be of interest to narrow this down to determine if AKAP79/150 is responsible for the effects on AIS enrichment observed in this study. Intriguingly, a close interplay between Kv7 CaM binding modes and AKAP79/150 binding has been suggested raising the possibility that the two proteins co-operate to regulate AIS localization (Zhang and Shapiro, 2016).

The second domain identified had the opposing impact on AIS localization as the deletion of helix D reduced the AIS enrichment of Kv7.3 chimeras. This effect was associated with an increased FRAP phenotype with the largest effect observed in the chimera that also lacks the proximal domain [SEP-TAC-Kv7.3(651–872)]. The increased fluorescence recovery observed in SEP-TAC-Kv7.3(651–872) as compared to SEP-TAC-Kv7.3CTΔhelixD is unclear, but could be related to chimera assembly as SEP-TAC-Kv7.3(651–872) lacks helix C. The FRAP phenotype of chimeras lacking helix D could suggest a significant impact of this domain on channel anchoring in the AIS. In support of this, deletion of helix D largely eliminated the nanoscale periodic organization observed for SEP-TAC chimeras containing the domain. Intriguingly, helix D appears to confer AIS stability by a mechanism that works in addition to ankG as chimeras in which both helix D and the ankG binding motif were disturbed displayed an additive FRAP effect.

The effect of deleting helix D upon AIS localization and stability was reproduced in full-length Kv7 channels. We interestingly observed AIS localization of Kv7 channels in which the Kv7.3 helix D was deleted. However, similar to our observations on the C-terminal chimeras, Kv7.2 + SEP-TAC-Kv7.3ΔhelixD expression levels at the AIS were decreased as compared to the wild-type channels, which is in accordance with the previously published reductions in current densities (Schwake et al., 2006; Nakajo and Kubo, 2008). Since Kv7.2/Kv7.3 channels require co-assembly to localize to the AIS, our data would suggest that though helix D can confer assembly specificity, it is not required for channel assembly, but is rather permissive or promoting channel assembly. This has previously been reported for the Kv T1 assembly domain found in other Kv channel families (Tu et al., 1996; Kobertz and Miller, 1999; Zerangue et al., 2000). Our double-FRAP and full-length data would, however, be consistent with the helix D coiled-coil structure promoting stability to Kv7 channels at the AIS. It could be speculated that the assembled helix D coiled-coil forms the interface for interaction with a protein promoting AIS stability. The observation that the helix D mutant does not confer a dominant phenotype over the wild-type C-terminal tail in our double-FRAP experiments could be explained by the C-terminal chimeras displaying transient rather than stable interactions, which has previously been reported for TAC protein fused to Kv7.2 helices A-D (Alaimo et al., 2017). This would allow the C-terminal tail to transiently form coiled-coil structures with other wild-type C-terminal tails.

The C-terminal helix D mutant also demonstrated significant off-target expression in the distal axon and to a lesser extent on the soma and dendrites. The missing off-target removal of SEP-TAC-Kv7.3CTΔhelixD could implicate helix D in Kv7 endocytosis. Indeed, in the full-length setting helix D regulates Kv7.2/Kv7.3 surface expression and the Kv7.3 helix D has been reported to have a negative impact on Kv7.2/Kv7.3 current expression in the absence of the Kv7.2 helix D (Schwake et al., 2006; Nakajo and Kubo, 2008). Altogether, these observations suggest that helix D also regulates trafficking.

Finally, our data provide new insight into the ankG binding motif. The requirement of this motif for AIS localization and AIS stability of Kv7.2/Kv7.3 channels has been demonstrated in several studies (Chung et al., 2006; Pan et al., 2006; Rasmussen et al., 2007; Benned-Jensen et al., 2016). Our data support that this motif is critical for efficient AIS localization as its mutation resulted in a non-polarized localization of both CD4 and SEP-TAC- chimeras. Its importance is further emphasized by the increased fluorescence recovery of chimeras with a mutated ankG binding motif and by the significant rescue of this FRAP phenotype by co-expression with chimeras containing an intact ankG binding motif. Interestingly, the FRAP phenotype of chimeras containing the wild-type C-terminal tail was unaffected by the presence of the ankG binding mutants indicating that while the presence of an ankG binding motif is required, the number of ankG binding motifs is not important for stability.

In summary, we have identified two motifs in the C-terminus of Kv7.3 channels with opposing impact on AIS localization efficiency of CD4 and SEP-TAC chimeras. A MP motif decreases AIS localization while helix D increases it. The fact that the identified motifs regulate efficiency but are not required for AIS localization could indicate they play a role in fine-tuning Kv7 channel numbers at the AIS. Future studies should determine the protein interaction partners of the MP and helix D domains.

## Data Availability Statement

All datasets generated for this study are included in the article/supplementary material.

## Ethics Statement

This study was reviewed and approved by the Department of Experimental Medicine at the University of Copenhagen, or performed in accordance to the Animal Welfare Law of the Federal Republic of Germany (Tierschutzgesetz der Bundesrepublik Deutschland, TierSchG) and the regulations given in §4 TierSchG. Ethical review and approval was not required for tissue preparation because euthanizing rodents for subsequent tissue preparation is not required as per local legislations. Tissue preparation was performed in both Denmark (approved by The Department of Experimental Medicine at the University of Copenhagen, Denmark) and Germany (following the Animal Welfare Law of the Federal Republic of Germany). In both countries no specific authorization is required for euthanizing animals.

## Author Contributions

LH, ED’E, EA, and HR designed the research, analyzed, and interpreted the data. LH, ED’E, and HR prepared the manuscript. All authors approved the final version of the manuscript and performed experimental work.

## Conflict of Interest

The authors declare that the research was conducted in the absence of any commercial or financial relationships that could be construed as a potential conflict of interest.
